# Investigating Multiple Mediators to Mitigate Socioeconomic Differences in Patient‐Reported Outcomes After Stroke: A Nationwide Register‐Based Study

**DOI:** 10.1161/JAHA.124.039466

**Published:** 2025-02-19

**Authors:** Anita Lindmark, David Darehed

**Affiliations:** ^1^ Department of Statistics, Umeå School of Business, Economics and Statistics Umeå University Umeå Sweden; ^2^ Department of Public Health and Clinical Medicine, Sunderby Research Unit Umeå University Umeå Sweden

**Keywords:** low socioeconomic status, mediation analysis, patient‐reported outcome measures, risk factors, stroke, Epidemiology, Risk Factors, Cerebrovascular Disease/Stroke, Disparities

## Abstract

**Background:**

Substantial socioeconomic differences in patient‐reported outcome measures (PROMs) 3 months after stroke have recently been shown. We aimed to understand the underlying mechanisms and investigate potential interventional targets to equalize differences.

**Methods:**

All patients aged 18 to 64 years, independent in activities of daily living, registered with a first‐time stroke in Riksstroke (the Swedish Stroke Register) from 2015 to 2017 were included. PROMs 3 months after stroke included activities of daily living status, mood, fatigue, pain, and general health. Socioeconomic status (SES) was measured on the basis of income and education. Using causal mediation analysis, we simulated the effect of interventions on the distributions of smoking, metabolic health (diabetes, antihypertensive treatment, statin treatment), atrial fibrillation, and stroke characteristics (stroke type, severity) on the absolute SES‐related risk difference in PROMs.

**Results:**

Of 6910 patients, 8% had become dependent in activities of daily living, 13% reported low mood, 42% fatigue, 23% pain, and 17% poor general health 3 months after stroke. Adjusted for sex and age, low SES was associated with increased absolute risks of poor PROMs with between 6% and 18% compared with higher SES with the largest increase for general health (18.2% [95% CI, 13.5%–22.9%]). Intervening to shift the distribution of all mediators among patients with low SES to those of patients with higher SES potentially reduces SES disparities by a proportion of 14% to 45%. For most PROMs the most important intervention was reducing smoking and improving metabolic health.

**Conclusions:**

Working‐age patients with low SES report more severe outcomes 3 months after stroke than patients with higher SES. Targeted interventions reducing the prevalence of smoking, diabetes, hypertension, and high cholesterol in patients with low SES could mitigate these disparities.

**Clinical Perspective**

**What Is New?**

- Patient‐reported outcomes after stroke including dependency in activities of daily living, pain, fatigue, depression, and general health among working‐age patients differs significantly between socioeconomic groups, with absolute risk increases of a poor outcome ranging between 6% and 18% among patients with low socioeconomic status (SES) compared with more privileged groups.
- After adjustment for multiple mediators including smoking, metabolic health, atrial fibrillation, and stroke characteristics, this study shows that shifting the distributions of mediators among patients with low SES to those of patients with higher SES potentially could reduce SES disparities in patient‐reported outcomes by a proportion of 14% to 45%.
- The most important interventional targets aiming to equalize patient‐reported outcomes between socioeconomic groups would be to reduce the prevalence of smoking, hypertension, diabetes, and hypercholesterolemia among patients with low SES.

**What Are the Clinical Implications?**

- Health care interventions aiming to improve patient‐reported outcomes after stroke should focus on smoke cessation and improving metabolic health among patients with low SES.
- Although our findings show that mediators play a crucial role in patient‐reported outcomes after stroke among patients of different socioeconomic groups, a significant part of the disparity remains unexplained, and hence further research is warranted to find other potentially effective interventional targets.

**Nonstandard Abbreviations and Acronyms**
NIHSS: National Institutes of Health Stroke Scale
PROMs: patient‐reported outcome measures
Riksstroke: Swedish Stroke Register

Previous studies have shown that low socioeconomic status (SES) is associated with an increased risk of a multitude of negatives regarding stroke, including an increased risk of stroke, higher mortality rate, more disability, more severe strokes, less access to care, lower quality of care, and inferior secondary prevention after stroke.^1^, ^2^, ^3^, ^4^, ^5^, ^6^, ^7^ Patient‐reported outcome measures (PROMs) in relation to SES after stroke are underused, as most studies have focused on survival or clinician‐reported outcomes. However, a recent study showed considerable differences in PROMs after stroke, with lower mood and more disability, fatigue, and pain for patients with low SES.^8^ These differences were particularly pronounced among working‐age patients.

Although the underlying mechanisms of the negative effects of low SES on stroke have remained unknown, recent studies using mediation analyses are now advancing our understanding of these complex causal relationships, revealing new possible interventional targets.^9^ This is exemplified by previous studies, which have shown that a large part of income‐related inequalities in the 3‐month mortality rate^10^ and long‐term disability^11^ after stroke is mediated through stroke severity, while quality of acute care did not mediate the effect of SES on the 30‐day mortality rate and readmission.^12^ Using novel causal mediation analysis methods allowing the evaluation of multiple mediators, it was found that almost one third of the association between low education and severe stroke was explained by additional risk factors, making these risk factors an obvious target for clinical interventions aiming to reduce stroke severity.^13^ Another recent study evaluating multiple mediators showed that hypothetical interventions on SES‐related differences in primarily comorbidity and stroke severity could potentially prevent up to 40 of every 1000 patients with low SES from becoming dependent or dying following their stroke.^14^

In this study, we aim to investigate causal mechanisms behind and identify potential interventional targets to mitigate SES differences in PROMs 3 months after stroke among working‐age patients. Since cardiovascular risk factors and stroke characteristics have previously been shown to play an important role in SES differences in survival,^14^ we want to investigate whether these are also of importance for socioeconomic disparity in PROMs.

## Methods

This research is covered by ethical approval from the regional ethics review board in Umeå, Sweden (reference number 2017/184‐31). Patients are informed about registration in Riksstroke (the Swedish Stroke Register), and are offered the right to decline participation (opt‐out consent). Given the sensitive nature of the data gathered for this study, qualified researchers with training in human subject confidentiality protocols can request access to the data set. These requests are subject to ethical approval and permission from Riksstroke and Statistics Sweden and should be directed to Riksstroke at riksstroke@regionvasterbotten.se.

### Study Design and Population

This is a cohort study based on prospectively collected national register data. The main data source was Riksstroke, merged with information on SES retrieved from the Longitudinal Integrated Database for Health Insurance and Labor Market Studies, which is managed by Statistics Sweden, who linked the data on the patient‐level using the Swedish national identification numbers. The researchers were supplied a pseudonymized data set.

Riksstroke is a quality register aiming to watch and support improvements of in‐hospital stroke care in Sweden. The register includes all 72 Swedish hospitals providing acute stroke care and covers >90% of all patients with acute stroke.^15^ In addition to data registered by hospital staff at the acute stage, information is gathered at follow‐up 3 and 12 months after stroke through questionnaires administered by the hospitals but filled out by the patients.^16^ The 3‐month follow‐up is performed via mail, telephone, or in conjunction with health care visits and during the study period 87% to 88% of questionnaires were filled out by the patients either by themselves or with the aid of another person.

**Figure 1:**
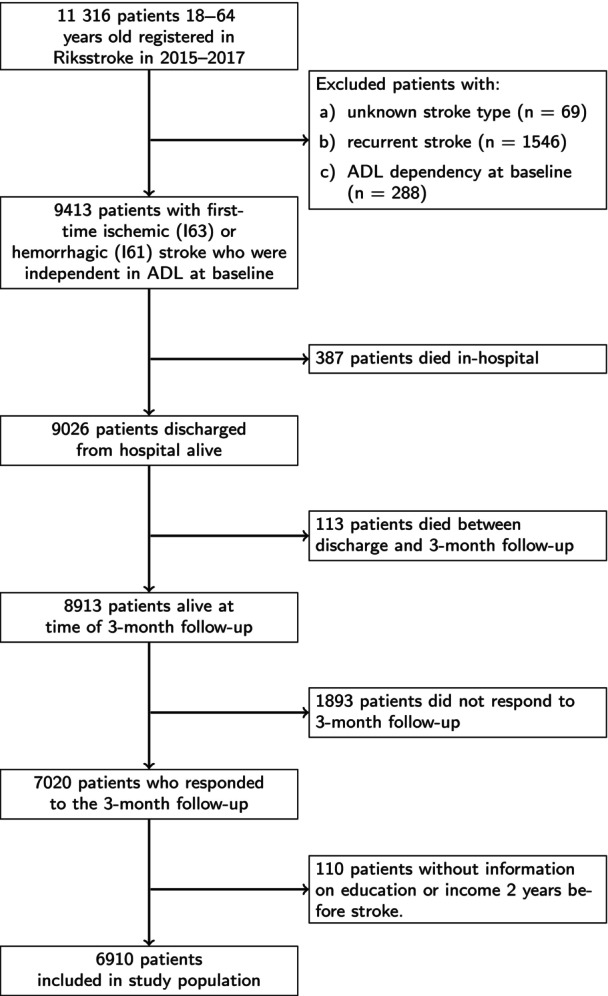
Study flowchart. ADL indicates activities of daily living; and Riksstroke, Swedish Stroke Register.

The inclusion criteria for the current study were that the patient was registered with a first‐time ischemic (*International Classification of Diseases, Tenth Revision* [*ICD‐10*] code I63) or hemorrhagic (I61) stroke in Riksstroke in 2015 to 2017, was aged 18–64 years and independent in activities of daily living (ADL) at the time of stroke, and provided follow‐up information at 3 months (see Figure 1 for the study flowchart).

This study is reported in adherence with the Reporting of Studies Conducted Using Observational Routinely Collected Health Data guidelines.^17^

### Variables

**Figure 2:**
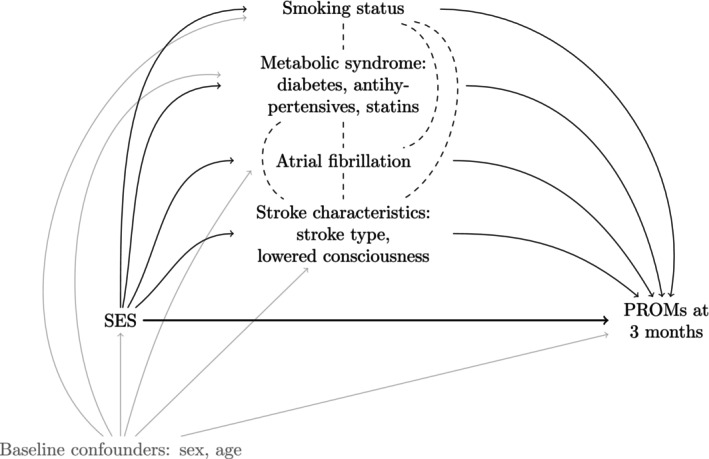
A directed acyclic graph illustrating the hypothesized relationships between the variables in the study. Arrows show an assumed causal relationship while dashed lines show associations without an assumption of directionality. PROMs indicates patient‐reported outcome measures; and SES, socioeconomic status.

The hypothesized relationships between the study variables are visualized in Figure 2. We assume that there are potential causal pathways via the mediators between the SES exposure and the patient‐reported outcomes at 3 months. However, as the mediators are measured at the same point in time, we do not assume any directionality in the relationships between mediators. The variable definitions are outlined below.

#### Outcome: PROMs at 3 Months

Five binary outcome variables based on PROMs recorded at the Riksstroke 3‐month follow‐up were investigated: self‐rated general health, ADL dependency, and experience of low mood, fatigue, and pain.

To better capture disability, ADL dependency at 3 months after stroke was analyzed as a composite measure, defined by the patient being unable to manage dressing, using the bathroom, or moving around indoors unassisted. Low mood, fatigue, and pain were measured using the questions “Do you feel depressed?,” “Do you feel tired?,” and “Do you have any pain?,” with response alternatives “never or almost never”/sometimes/often/constantly/“do not know.” Self‐rated general health was measured using the question “How would you assess your general health?,” with response alternatives “very good”/“quite good”/“quite poor”/“very poor”/“do not know.” These PROMs were each dichotomized into “poor outcome” versus “good outcome,” where poor outcome was defined, respectively, as experiencing low mood, fatigue, and pain often or constantly, and rating their general health as quite poor or very poor. The response alternative “do not know” was treated as missing. The Riksstroke PROM questions have been validated against established instruments.^14^

#### Exposure: SES

For the exposure SES we used a composite measure based on the patient's highest attained education level (primary school, secondary school, or university) and the patient's portion of the family's disposable income 2 years before the stroke. The patient's portion of the family's disposable income takes into account the size and composition (number of adults and children) of the household to estimate the portion of the total income of the household that is disposed by each individual.^18^ We defined the income levels on the basis of year‐specific income tertiles, and to capture the individual position in the peer group, we calculated these from all patients aged 18 to 64 years registered in Riksstroke in 2015 to 2017 (ie, not limited to patients with first‐time stroke independent in ADL). Low income was defined as income in the lowest tertile, high income as income in the highest tertile, and mid income as income in the middle tertile. We used a 3‐tiered categorization of SES^14^: low (primary school education and income in the lowest tertile), high (university education and income in the highest tertile), and mid (everyone not in the low or high categories).

#### Mediators and Baseline Confounders

The mediators/mediator groups investigated were smoking habits (dichotomized as smoker versus nonsmoker or unknown), metabolic health, atrial fibrillation (yes/no), and stroke characteristics. The mediator group metabolic health consisted of 3 variables: whether the patient had diabetes and whether the patient was prescribed antihypertensives or statins at the time of stroke, where the latter 2 serve as proxies for hypertension and hypercholesteremia. The mediator group stroke characteristics consisted of 2 variables: stroke type (hemorrhagic versus ischemic stroke) and lowered consciousness at hospital arrival (reaction‐level scale >1). The mediators were all registered at the time of stroke.

Baseline confounders were sex (male/female) and the age of the patient at the time of stroke. For regression models, age was centered around its mean. All mediators and baseline confounders were chosen before the study, and the choice was based on medical expertise and availability in the registers.

### Statistical Analysis

We present patient characteristics separated by SES and for the whole study population as median and interquartile range for continuous variables and frequencies and proportions for categorical variables.

Preliminary analyses of the associations in Figure 2 were performed using logistic regression analyses of the associations between SES and poor outcome in each PROM, between SES and each mediator, and between each mediator and poor outcome in each PROM. All models were adjusted for sex and age, and associations are presented as odds ratios (ORs) with 95% CIs.

We performed a sensitivity analysis of level of consciousness as a proxy for stroke severity by fitting outcome models for those patients (around 60%) with both level of consciousness and the National Institutes of Health Stroke Scale (NIHSS) measured to see the impact of adding NIHSS on the estimated coefficients.

#### Interventional Effects

To evaluate the reductions in SES disparities in PROMs that could potentially be achieved through interventions targeting the mediators, we used an approach that has been suggested for settings where there is no available data on well‐defined interventions.^19^ This approach focuses on evaluating the potential impact of hypothetical interventions through simulations, which can give a better understanding of the causal mechanisms at play and possibly serve as a basis for policymakers when deciding on the most appropriate targets for implementation of actual interventions.

In our study, we investigate hypothetical interventions to change the levels of mediators in patients with low SES to be as those of patients with higher SES and thereby eliminate SES differences in PROMs that are due to differences in the mediators. We define *interventional effects* on the basis of the following hypothetical interventions (for more detailed definitions, see Data S1):

1. Jointly intervening to change the levels of all mediators in patients with low SES to those of patients with mid or high SES.
2. Intervening to reduce the level of smoking in patients with low‐SES to be as in patients with mid or high SES, independently of the other mediators.
3. Intervening to jointly change the levels of the mediators in the metabolic health group (ie, reduce the prevalence of diabetes and need for antihypertensives and statins) in patients with low SES to be as in patients with mid or high SES, independently of the other mediators.
4. Intervening to change the level of atrial fibrillation in patients with low SESto be as in patients with mid or high SES, independently of the other mediators.
5. Intervening to jointly change the levels of the mediators in the stroke characteristics group (ie, change the balance between ischemic and hemorrhagic stroke and reduce lowered consciousness) in patients with low SES to be as in patients with mid or high SES, independently of the other mediators.

For each of these interventions, we examine the potential *reduction* in the average (confounder adjusted) total absolute risk difference in poor PROMs for patients with low SES versus patients with mid and high SES, as well as the *remaining disparity* that would not be eliminated by the intervention. In mediation analysis terms, the reductions in total risk from mediator interventions correspond to indirect effects and the remaining disparities to direct effects. We look at 2 mediator level shifts, from levels in low SES to levels in mid SES and from levels in low SES to levels in high SES.

For estimation, we adapted a simulation‐based procedure for interventional effects with multiple mediators,^20^ as described in Moreno‐Betancur et al^19^ to our setting with both single mediators and mediator groups. This approach has also been used to estimate other interventional effects with multiple mediators.^20^, ^21^ Here, we briefly summarize the procedure; more details are provided in Data S1, and analysis code is available on GitHub. In short, we constructed logistic regression models for each dichotomized PROM given SES, mediators, and baseline confounders, as well as for each mediator, given subsets of “preceding” mediators, SES, and baseline confounders. To reduce model misspecification bias, we included all SES–mediator and mediator–mediator interactions as well as age squared to make the models flexible. We then simulated mediator values from these models corresponding to different intervention scenarios and predicted the risk of poor PROMs on the basis of the simulated mediators for each patient. For each of 500 simulation replications, the effects were obtained by contrasting average predicted risks across all patients, and finally averaged across all simulations. To estimate the standard errors, we used bootstrap with 1000 bootstrap replications. The effect estimates are presented as absolute risk differences with 95% CIs.

Since the estimation combines Monte Carlo simulation and bootstrap, it is computationally intensive, and rather than add multiple imputation steps to the procedure, we opted to use a complete case analysis approach to missing values. This means that for each analysis we excluded those patients who were missing information on any of the variables of interest in that analysis.

All analyses were performed using R Statistical Software (R Foundation for Statistical Computing, Vienna, Austria),^22^ and the results are reported in accordance with the Guideline for Reporting Mediation Analyses statement.^23^

## Results

A total of 8913 patients met the initial inclusion criteria for the study, of which the 6910 patients (77.5%) who had information on the SES variables and responded to the follow‐up were included in the study (Figure 1). Comparing responders with nonresponders (Table S1), the latter had higher proportions of low SES, male sex, smoking, diabetes, hemorrhagic stroke, and lowered consciousness but were younger and had fewer prescribed statins.

In the study population, 64.5% were men (Table), and the median age was 57 years (interquartile range, 51–61). The majority was in the mid‐SES group, with 546 patients (7.9%) in the low‐SES group and 1014 (14.7%) in the high‐SES group.

**Table 1:** Descriptive Statistics Separated by SES and for the Whole Study Population

	Low SES (n=546)	Mid SES (n=5350)	High SES (n=1014)	Overall (n=6910)
Sex, n (%)				
Male	325 (59.5)	3488 (65.2)	642 (63.3)	4455 (64.5)
Female	221 (40.5)	1862 (34.8)	372 (36.7)	2455 (35.5)
Age, y, median (quartiles 1–3)	57.5 (51.0–62.0)	57.0 (50.0–61.0)	58.0 (52.0–62.0)	57.0 (51.0–61.0)
Mediators (at time of stroke)				
Smoking status, n (%)				
Smoker	247 (45.2)	1424 (26.6)	89 (8.8)	1760 (25.5)
Nonsmoker	253 (46.3)	3557 (66.5)	852 (84.0)	4662 (67.5)
Unknown	46 (8.4)	369 (6.9)	73 (7.2)	488 (7.1)
Diabetes, n (%)				
Yes	140 (25.6)	825 (15.4)	117 (11.5)	1082 (15.7)
Missing	0 (0)	3 (0.1)	1 (0.1)	4 (0.1)
Antihypertensives, n (%)				
Yes	250 (45.8)	1950 (36.4)	322 (31.8)	2522 (36.5)
Missing	2 (0.4)	14 (0.3)	0 (0)	16 (0.2)
Statins, n (%)				
Yes	104 (19.0)	823 (15.4)	136 (13.4)	1063 (15.4)
Missing	1 (0.2)	13 (0.2)	2 (0.2)	16 (0.2)
Atrial fibrillation, n (%)				
Yes	48 (8.8)	484 (9.0)	91 (9.0)	623 (9.0)
Missing	6 (1.1)	23 (0.4)	6 (0.6)	35 (0.5)
Stroke type, n (%)				
Hemorrhagic	69 (12.6)	717 (13.4)	142 (14.0)	928 (13.4)
Ischemic	477 (87.4)	4633 (86.6)	872 (86.0)	5982 (86.6)
Lowered consciousness, n (%)				
Yes	59 (10.8)	390 (7.3)	64 (6.3)	513 (7.4)
Missing	8 (1.5)	60 (1.1)	9 (0.9)	77 (1.1)
Outcomes (3 mo after stroke)				
ADL dependency, n (%)				
Yes	78 (14.3)	436 (8.1)	62 (6.1)	576 (8.3)
Missing	13 (2.4)	66 (1.2)	8 (0.8)	87 (1.3)
Low mood, n (%)				
Often/constantly	130 (23.8)	693 (13.0)	74 (7.3)	897 (13.0)
Missing	37 (6.8)	202 (3.8)	28 (2.8)	267 (3.9)
Fatigue, n (%)				
Often/constantly	265 (48.5)	2279 (42.6)	350 (34.5)	2894 (41.9)
Missing	20 (3.7)	140 (2.6)	17 (1.7)	177 (2.6)
Pain, n (%)				
Often/constantly	167 (30.6)	1280 (23.9)	153 (15.1)	1600 (23.2)
Missing	38 (7.0)	181 (3.4)	20 (2.0)	239 (3.5)
General health, n (%)				
Poor/very poor	149 (27.3)	910 (17.0)	118 (11.6)	1177 (17.0)
Missing	53 (9.7)	312 (5.8)	32 (3.2)	397 (5.7)

ADL indicates activities of daily living; and SES, socioeconomic status.

The proportion of male patients was smaller in the low‐SES group (59.5%), while the age distributions were similar in all 3 SES groups. For the mediators, there was a strong SES gradient for smoking with decreasing proportions of smokers with increasing SES. The proportions of diabetes, antihypertensive medication, statins, and lowered consciousness were largest in the low‐SES group, while differences in atrial fibrillation and stroke type were small between SES groups (Table).

Proportions reporting poor PROMs ranged from 8.3% (ADL dependency) to 41.9% (fatigue). A distinct SES gradient was found for all PROMs, with decreasing proportions of patients reporting poor outcomes with increasing SES (Table).

Smoking status was unknown for 7.1% of all patients and was included in the reference category no/unknown in the analyses. Missing information on the other mediators was small, ranging from 0.1% to 1.1%, with the largest proportion for lowered consciousness (Table). For the PROMs, the proportions missing ranged from 1.3% for ADL dependency to 5.7% for general health. Generally, the proportions missing were larger for patients with low SES.

### Preliminary Analyses: Logistic Regression Models

Adjusted for the baseline confounders sex and age (age and age squared), low SES was associated with increased odds of a poor outcome for all PROMs, both compared with mid and high SES but with a more pronounced increase for the latter (Table S2, Exposure–Outcome Models, Model 1). These associations were attenuated but largely remained with additional adjustment for mediators (Table S2, Exposure–Outcome Model, Model 2).

Low SES was also associated with increased odds of smoking, diabetes, antihypertensive treatment, statin treatment, and lowered consciousness (Table S3). The association was especially pronounced for low versus high SES and smoking (OR, 8.75 [95% CI], 6.66–11.57). The associations between low SES and atrial fibrillation and hemorrhagic stroke were small in size and not statistically significant.

For the mediator–outcome associations (Table S2, Mediator–Outcome Models), smoking was associated with increased odds of a poor outcome independent of sex and age for all PROMs except ADL dependency. All 3 mediators in the metabolic health group were associated with increased odds of fatigue, pain, and poor self‐rated general health, while the associations were weaker for low mood. There were no associations between atrial fibrillation and experiencing fatigue and pain but a significant association with poor general health (OR, 1.31 [95% CI, 1.05–1.62]). Hemorrhagic stroke was associated with increased odds of ADL dependency (OR, 3.23 [95% CI, 2.64–3.94]) but not associated with any of the other PROMs. Lowered consciousness was associated with increased odds of low mood, pain, and, to a lesser extent, poor general health, and strongly associated with increased odds of ADL dependency (OR, 12.38 [95% CI, 10.01–15.30]).

### Interventional Effects

**Figure 3:**
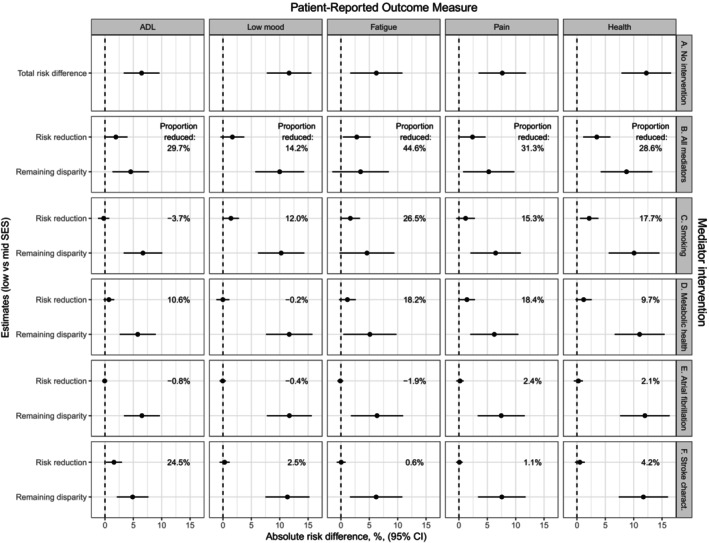
Estimated absolute risk differences in PROMs with 95% CIs for low versus mid SES, adjusted for sex, age, and age squared. Difference under (**A**), no mediator intervention (ie, total risk difference); (**B**) joint intervention on all mediators; (**C**) intervention on smoking status; (**D**) intervention on mediators in the metabolic health group; (**E**) intervention on atrial fibrillation; and (**C**) intervention on mediators in the stroke characteristics group. Dashed lines correspond to a risk difference of 0. Proportion reduced=(Risk reduction)/(Total risk difference)×100. PROMs indicates patient‐reported outcome measures; and SES, socioeconomic status.

**Figure 4:**
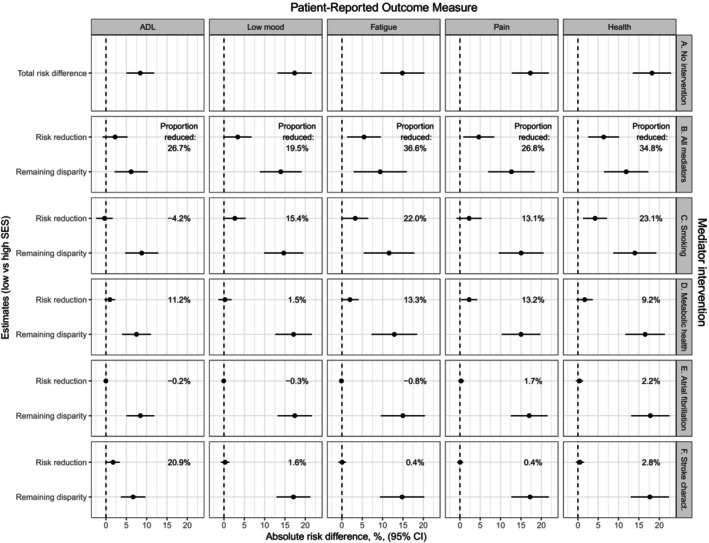
Estimated absolute risk differences in PROMs with 95% CIs for low versus high SES, adjusted for sex, age, and age squared. Difference under (**A**) no mediator intervention (ie, total risk difference); (**B**) joint intervention on all mediators; (**C**) intervention on smoking status; (**D**) intervention on mediators in the metabolic health group; (**E**) intervention on atrial fibrillation; and (**C**) intervention on mediators in the stroke characteristics group. Dashed lines correspond to a risk difference of 0. Proportion reduced=(Risk reduction)/(Total risk difference)×100. PROMs indicates patient‐reported outcome measures; and SES, socioeconomic status.

Results from the mediation analyses for all PROMs are summarized in Figure 3 (low versus mid SES) and Figure 4 (low versus high SES) with more detailed results for each PROM in Tables S4 through S8.

Focusing on the total absolute risk differences in poor outcomes between patients with low SES and patients with mid SES (Figure 3A) and high SES (Figure 4A), we observed increased risks for patients with low SES compared with more privileged patients for all PROMs after adjustment for sex and age. As expected, these differences were larger in size when contrasting patients with low versus high SES (between 8.4% and 18.2%) than when contrasting patients with low versus mid SES (6.2%–12.2%). The differences were smaller for ADL dependency (Table S4) and (for the low versus mid contrast) fatigue (Table S6), and larger for low mood (Table S5) and poor general health (Table S8).

Figures 3 and 4 summarize how much these differences could potentially be reduced by intervening to shift the mediator distributions among patients with low SES to those of mid SES (Figure 3) or patients with high SES (Figure 4), jointly (Figure 4B) or 1 at a time independently of the other mediators (Figure 4C through 4F).

Potential reductions in the total risk difference from jointly intervening to shift all mediator distributions were seen for all PROMs and for both SES contrasts (Figures 3B and 4B) but did not reach statistical significance at the 5% level for ADL (Table S4) or the low versus mid contrast for low mood (Table S5). For most PROMs, around 25% to 35% of the total risk differences could potentially be reduced from the joint intervention on all mediators, with smaller proportions for low mood (14.2% for low versus mid SES, 19.5% for low versus high SES) and larger proportions for fatigue (44.6% for low versus mid SES and 36.6% for low versus high SES).

For the interventions focusing on 1 mediator/mediator group at a time, similar patterns were seen for low mood, fatigue, and poor general health, where intervening to shift the distribution of smoking status (Figures 3C and 4C) gave the largest risk reduction for both SES contrasts. For low mood (Table S5), none of the other mediator shifts had any impact, while reductions in fatigue, pain, and poor health were seen from jointly shifting the mediator distributions in the metabolic health group (Tables S6 through S8, Figures 3D and 4D) but reached statistical significance only for pain. Interventions focusing on atrial fibrillation (Figures 3E and 4E) and stroke characteristics (Figures 3F and 4F) were not associated with any risk difference reductions. The pattern differed for ADL dependency, with by far the largest potential reduction in risk difference coming from intervening to shift the distributions of stroke characteristics (Table S4, Figures 3F and 4F).

### Sensitivity Analysis Level of Consciousness

The mean and median levels of NIHSS were higher for patients with lowered or missing level of consciousness compared with fully conscious patients (Table S9). Overall, 39.3% of patients were missing NIHSS, with considerably higher proportions among those with lowered or missing level of consciousness.

Estimated outcome models with and without adjustment for NIHSS among patients with both lowered consciousness and NIHSS observed are presented in Table S10. For low mood, fatigue, pain, and general health, the estimated ORs for SES and mediators other than level of consciousness were unchanged or changed very little with the addition of NIHSS. For ADL dependency, associations with SES and diabetes were stronger with additional adjustments, while the associations with atrial fibrillation and hemorrhagic stroke were attenuated.

## Discussion

In this nationwide, register‐based study we explained causal mechanisms behind previously identified SES disparities in PROMs 3 months after stroke in working‐age (18‐ to 64‐year‐old) patients with stroke^8^ and identified some potential interventional targets to mitigate differences. We found considerable differences in the absolute risk of poor PROMs comparing patients with low SES with patients with mid or high SES. Intervening on all mediators (smoking status, metabolic health, atrial fibrillation, and stroke characteristics) jointly was estimated to potentially reduce SES disparities by 14% to 45%, with the lowest proportions for low mood and the highest for fatigue.

For the separate mediator/mediator group interventions, the pattern was different for ADL dependency compared with the other PROMs. ADL dependency is, compared with the other PROMs in this study, more directly connected to neurological deficits such as motor and cognitive impairment, and hence it is not surprising that level of consciousness and type of stroke have a greater impact on this outcome. Surprisingly, level of consciousness and stroke type only had minor impact on mood, fatigue, pain, and general health, which all can be severely affected by a stroke and to a high degree reflect perceived deficits after stroke. Instead, we found that smoking status and to a lesser extent diabetes, antihypertensive treatment, and statin treatment was more important for these outcomes. SES differences in atrial fibrillation were small in this age group and interventions on the atrial fibrillation distribution alone had no impact on the SES disparities in poor PROMs. We considered including prescribed anticoagulants as a possible intervention target, but the number receiving this treatment was small (3.5% overall), making it difficult to study for this age group.

Translated into number of patients out of 1000, the estimated effects of intervening to shift the distribution of smoking status among patients with low SES to be as that of patients with high SES correspond to potentially preventing 27 from experiencing low mood, 33 from experiencing fatigue often or constantly, 23 from experiencing pain often or constantly, and 42 from experiencing poor general health.

The causal mechanisms involved in the relationship between SES and PROMs after stroke have never been investigated previously. It is interesting to note that the magnitudes of our results are in line with our earlier studies exploring direct and indirect effects of SES on poststroke outcomes. A previous study on the causal pathway between SES and ADL dependency and death 3 months after stroke showed that up to 40 of every 1000 patients could be saved from dying or becoming ADL dependent if SES differences in acute care, comorbidity, and stroke severity could be reduced.^14^ Another study showed that almost one third of the association between low SES and the risk of a severe stroke was explained by risk factors including diabetes, previous stroke, and ADL dependency.^13^

A major strength of our study is the nationwide scope including all hospitals caring for patients with stroke in Sweden, which, combined with the high national coverage, vouches for an unselected population at the baseline data collection. Other strengths include the recurrent validations of the register, the prospective nature of the data collection, and the use of novel methods for causal mediation analysis.^15^

Our simulations show that a large part of SES disparities in PROMs at 3 months after stroke would remain even if we could intervene on the mediators studied. More research is needed to find other potential effective targets for interventions. Since smoking status was 1 of the single most important mediators, it would be of interest to investigate other lifestyle factors including diet, physical activity, and alcohol consumption. However, we were limited to the variables available in the registers. It would have been interesting to analyze the mechanisms at play separately for ischemic and hemorrhagic strokes, but the relatively few hemorrhagic strokes did not allow this. In future work on a larger data set, it is also of interest to investigate sex‐related differences in potential intervention targets. The data's being from 2015 to 2017 means that thrombectomy was not as common as it is today, which, combined with thrombolysis, is a mediator that could be considered for inclusion in future studies.

We also had limited information on comorbidities and used information on prescribed antihypertensives and statins as proxies for hypertension and hypercholesteremia as we did not have access to clinical measurements of blood pressure and cholesterol levels. More objective measures based on neuroimaging would be preferrable, but such information is unfortunately not available in Riksstroke. We did have access to the NIHSS but only for around 60% of the patients, and hence as a proxy for stroke severity we used level of consciousness on the basis of the reaction‐level scale instead, which had only 1.1% missing data. Level of consciousness has been shown to be a good approximation of the full NIHSS for prediction of poststroke death.^24^ We performed a sensitivity analysis by fitting outcome models for patients with both level of consciousness and NIHSS observed and found similar results for most PROMs with and without NIHSS. There were some differences for ADL dependency that could indicate that the main analysis underestimates the SES differences and the effect via metabolic health and overestimate the effect via stroke type in our analysis. As in all observational studies, unobserved confounding cannot be ruled out.

The PROM questions in the Riksstroke follow‐up are simplified to keep the number of questions at a minimum and increase response rate. In validations against established instruments (Barthel Index, Beck Depression Inventory, Fatigue Symptom Inventory, Brief Pain Inventory Short Form, and the 12‐Item Short Form Survey), the PROMs used in Riksstroke were all shown to have high specificity (75%–100%).^25^ Sensitivity was shown to be high for ADL, pain, and fatigue (95%–98%) but modest for health and low mood (24%–38%). An important limitation is the lack of prestroke assessment of the PROMs, which means the results in the worst‐case scenario represent the patient's prestroke condition. However, it is not uncommon for stroke survivors to experience lasting symptoms, including poststroke fatigue, depression, and pain, and hence it is fair to believe that a considerable part of the poststroke assessment reflects the consequences of the stroke itself.

We used a composite measure of SES based on education and income to capture various aspects of SES. Education tends to be set earlier in life, while income varies over time,^26^ and combined measures have been shown to give more comprehensive estimates of health disparities.^27^ We did not, however, have access to data on occupation and therefore likely miss important aspects related to work environment and work‐related stress.^26^

The study results are based on patients (77.5%) who responded to the 3‐month follow‐up questionnaire and had information on the SES variables. Some differences in patient characteristics were seen between responders and nonresponders, with the most noteworthy difference being that nonresponders were almost twice as likely to have low SES. Among responders, the proportions of missing values were low, while nonresponders generally had slightly more missing values. Nonresponders were also found to be more likely to have lowered consciousness upon hospital arrival compared with responders; hence, it is possible that a poorer outcome could be the reason for nonresponse, meaning that SES differences are more likely underestimated rather than overestimated in our study. Since the estimation of the main results is computationally intensive, using multiple imputation was not practicable in the current study.

All things considered, we believe our results are robust and should be applicable to health care organizations like the Swedish health care system.

## Conclusions

Working‐age patients with low SES experience more severe outcomes 3 months after stroke than patients with higher SES. Targeted interventions to reduce the prevalence of smoking, diabetes, hypertension, and high cholesterol in low‐SES groups could significantly mitigate these disparities.

## Sources of Funding

The study was funded by Vetenskapsrådet (Swedish Research Council, grant 2018‐02670).

## Disclosures

None.

## Supporting information

Data S1
Tables S1–S10
