# Supplementary material for: Investigating Multiple Mediators to Mitigate Socioeconomic Differences in Patient‐Reported Outcomes After Stroke: A Nationwide Register‐Based Study
Source: J Am Heart Assoc. 2025 Feb 19;14(5):e039466. doi: 10.1161/JAHA.124.039466 (PMC12132664; doi:10.1161/JAHA.124.039466)
Supplement: Supplementary file 1 — Data S1 Tables S1–S10 [file JAH3-14-e039466-s001.pdf]

SUPPLEMENTAL MATERIAL

Data S1.

Supplemental Methods

1.1 Interventional effects

In this study we use an approach suggested by Moreno-Betancur et al.[19] to evaluate the effects of hypothetical interventions in the setting where we have multiple mediators of interest but no data on well-defined interventions that have already been implemented. Instead, effects are defined in terms of hypothetical interventions that could potentially be implemented to mitigate disparities.

In addition to investigating an intervention targeting all mediators (smoking status, “Metabolic health”, atrial fibrillation, and “Stroke characteristics”) jointly, we have adapted the “one-policy premise” approach described by Moreno-Betancur et al.[19] which focuses on interventions targeting only one single mediator at a time to our setting where we are interested in both interventions targeting single mediators (smoking status and atrial fibrillation, respectively) and interventions targeting groups of mediators jointly (mediators in the “Metabolic health” and “Stroke characteristics” groups, respectively).

The hypothetical interventions targeting the mediators are interventions to bring one or more mediators in the low SES group to the levels in the mid or high SES group (depending on the contrast of interest). This means shifting the distribution of the mediator or mediators of interest from that in the low SES group to that in the mid or high SES group.

In our study all mediators are measured at the same point in time, and we therefore want to avoid having to make assumptions regarding the causal ordering of the mediators, i.e. the directions of associations between them. Consequently, we adhere to the estimand assumptions E1-E3 in Moreno-Betancur et al.[19] when defining our hypothetical mediator interventions, i.e. for our study that:

1. intervening to shift the distribution of one mediator (or mediator group) does not have an impact on the distributions of the other mediators.
2. the intervention would shift the distribution of the single mediator (or mediator group) of interest in the low SES group to what it would be in the mid or high SES group (depending on the contrast of interest) given the confounders sex and age.
3. the intervention on a specific mediator (or mediator group) would (on average) remove the dependence between the specific mediator (mediator group) and the other mediators so that the joint distribution of the other mediators would remain at its level under low SES given the confounders sex and age.

For the intervention targeting all mediators jointly we assume that (E4 in Moreno-Betancur et al.[19]):

4. the intervention on all mediators jointly would shift the joint mediator distribution in the low SES group to what it would be in the mid or high SES group (depending on the contrast of interest) given the confounders sex and age.

In this study we aimed to estimate three types of effects:

- the *total absolute risk difference* in poor PROMs at 3 months after stroke for low SES patients versus mid or high SES patients, i.e. the disparity that we want to target through our mediator interventions.
- The *reduction in disparity,* i.e. in the total absolute risk difference, achieved by a specific mediator intervention. This contrasts the risk of poor PROMs in low SES patients without the intervention, i.e. with the joint mediator distribution of low SES patients, to the risk of poor PROMs in low SES patients with the intervention, i.e. shifting the distribution of the mediator (or mediators) of interest to be as that in mid or high SES patients.
- The *remaining disparity*, i.e. remaining total absolute risk difference, when implementing a specific mediator intervention. This contrasts the risk of poor PROMs in low SES patients with the intervention, i.e. shifting the distribution of the mediator (or mediators) of interest to be as that in mid or high SES patients, to the risk of poor PROMs in mid or high SES patients.

The specific effects corresponding to each mediator intervention investigated in the study are specified in the table on the next page, both conceptually and more concretely in terms of absolute risk differences.

To be able to estimate the effects based on the observed data (as outlined in Section 1.2) additional identification assumptions, such as no unobserved confounding, need to be made (see Moreno-Betancur et al.[19] for details).

Definitions of the effects of interest in the study. PROMs=Patient Reported Outcome Measures, TARD = Total Absolute Risk Difference, SES= Socioeconomic status.

**Effect**	**Conceptually**	**Absolute risk difference in poor PROMs (adjusted for sex and age) contrasting:**	
**Total absolute risk difference (TARD)**	Disparity in poor PROMs at 3 months after stroke that we want to target with our mediator interventions.	low SES patients vs.	mid (high)* SES patients
**Joint intervention on all mediators**			
Reduction in disparity (indirect effect)	Reduction of TARD from intervening to change the joint distribution of all the mediators in patients with low SES to be as that in patients with mid (high)* SES.	low SES patients with joint mediator distribution as that of low SES patients vs.	low SES patients with joint mediator distribution as that of mid (high)* SES patients
Remaining disparity (direct effect)	Remaining TARD when intervening to change the joint distribution of all the mediators in patients with low SES to be as that in patients with mid (high)* SES	low SES patients with joint mediator distribution as that of mid (high)* SES patients vs.	mid (high)* SES patients with joint mediator distribution as that of mid (high)* SES patients
**Intervention targeting only *smoking status***			
Reduction in disparity (indirect effect)	Reduction of TARD from intervening to change the distribution of smoking status in patients with low SES to be as in patients with mid (high)* SES, while the joint distribution of all the other mediators remains as in patients with low SES.	low SES patients with joint mediator distribution as that of low SES patients vs.	low SES patients with smoking status distribution as in mid (high)* SES patients and joint distribution of "metabolic health", atrial fibrillation, and "stroke characteristics" as in patients with low SES
Remaining disparity (direct effect)	Remaining TARD when intervening to change the distribution of smoking status in patients with low SES to be as in patients with mid (high)* SES, while the joint distribution of all the other mediators remains as in patients with low SES.	low SES patients with smoking status distribution as in mid (high)* SES patients and joint distribution of "metabolic health", atrial fibrillation, and "stroke characteristics" as in patients with low SES vs.	mid (high)* SES patients with joint mediator distribution as that of mid (high)* SES patients
**Intervention targeting only *"metabolic health"***			
Reduction in disparity (indirect effect)	Reduction of TARD from intervening to change the distribution of "metabolic health" in patients with low SES to be as in patients with mid (high)* SES, while the joint distribution of all the other mediators remains as in patients with low SES.	low SES patients with joint mediator distribution as that of low SES patients vs.	low SES patients with joint distribution of the mediators in the "metabolic health" group as in mid (high)* SES patients and joint distribution of smoking status, atrial fibrillation, and "stroke characteristics" as in patients with low SES

Remaining disparity (direct effect)	Remaining TARD when intervening to change the distribution of “metabolic health” in patients with low SES to be as in patients with mid (high)* SES, while the joint distribution of all the other mediators remains as in patients with low SES.	low SES patients with joint distribution of the mediators in the “metabolic health” group as in mid (high)* SES patients and joint distribution of smoking status, atrial fibrillation, and “stroke characteristics” as in patients with low SES	mid (high)* SES patients with joint mediator distribution as that of mid (high)* SES patients
**Intervention targeting only *atrial fibrillation***			
Reduction in disparity (indirect effect)	Reduction of TARD from intervening to change the distribution of atrial fibrillation in patients with low SES to be as in patients with mid (high) SES, while the joint distribution of all the other mediators remains as in patients with low SES.	low SES patients with joint mediator distribution as that of low SES patients vs.	low SES patients with atrial fibrillation distribution as in mid (high) SES patients and joint distribution of smoking status, “metabolic health”, and “stroke characteristics” as in patients with low SES
Remaining disparity (direct effect)	Remaining TARD when intervening to change the distribution of atrial fibrillation in patients with low SES to be as in patients with mid (high) SES, while the joint distribution of all the other mediators remains as in patients with low SES.	low SES patients with atrial fibrillation distribution as in mid (high) SES patients and joint distribution of smoking status, “metabolic health”, and “stroke characteristics” as in patients with low SES	mid (high) SES patients with joint mediator distribution as that of mid (high) SES patients
**Intervention targeting only *“stroke characteristics”***			
Reduction in disparity (indirect effect)	Reduction of TARD from intervening to change the distribution of “stroke characteristics” in patients with low SES to be as in patients with mid (high) SES, while the joint distribution of all the other mediators remains as in patients with low SES.	low SES patients with joint mediator distribution as that of low SES patients vs.	low SES patients with joint distribution of the mediators in the “stroke characteristics” group as in mid (high) SES patients and joint distribution of smoking status, metabolic health, and atrial fibrillation as in patients with low SES
Remaining disparity (direct effect)	Remaining TARD when intervening to change the distribution of “stroke characteristics” in patients with low SES to be as in patients with mid (high) SES, while the joint distribution of all the other mediators remains as in patients with low SES.	low SES patients with joint distribution of the mediators in the “stroke characteristics” group as in mid (high) SES patients and joint distribution of smoking status, metabolic health, and atrial fibrillation as in patients with low SES	mid (high) SES patients with the joint mediator distribution as that of mid (high) SES patients

* Depending on the contrast of interest, i.e. low vs. mid SES or low vs. high SES.

1.2 Estimation procedure

To estimate the effects, we adapted a Monte Carlo simulation approach previously used in the context of interventional effects with multiple mediators[19-21] to our setting. Code for the analyses can be found on GitHub[1]. To simplify the description, we introduce the following notation:

M1: smoking status (smoker vs. non-smoker or unknown).

M2: mediators in the "metabolic health" group, in the sequence: diabetes (yes/no), prescribed antihypertensives (yes/no), and prescribed statins (yes/no).

M3: atrial fibrillation (yes/no)

M4: mediators in the "stroke characteristics" group, in the sequence: stroke type (hemorrhagic vs. ischemic stroke) and lowered consciousness at hospital arrival (yes/no).

Note that the ordering among mediators as well as within mediator groups is not meant to reflect a causal ordering but is necessary for estimation of the joint mediator distributions.

The goal is to estimate the effects of shifts in mediator distributions that correspond to the hypothetical interventions of interest, adjusting for the confounders sex and age.

The mediator distributions are estimated through predicted probabilities from logistic regression models (Step A below) and the shifts are accomplished by drawing mediator values based on these predicted probabilities under specific intervention scenarios (Step B below).

These randomly drawn values are then used in logistic regression models for poor PROMs to predict risks of poor outcome for each PROM under each specific intervention scenario (Step B below). Finally, the effects of interest are given by contrasting these predicted risks under different scenarios (Step C below).

Step A: Model fitting:

In this step logistic regression models are fitted for the mediators and the PROMs based on the observed data. The regression models include all relevant SES-mediator and mediator-mediator interactions as well as age-squared. The models will be used for randomly drawing mediator and PROMs values in Step B.

1. M1 (smoking status) model:
    a. Model fitted for M1 *given only SES and confounders*

2. **M2** ("metabolic health") models:
    a. Models fitted for each of the mediators in **M2** *given the SES and confounders, and any mediator(s) in **M2** that precede in the sequence.*
    b. Models fitted for each of the mediators in **M2**, *given SES, confounders, any mediator(s) in **M2** that precede in the sequence, and M1.*

3. M3 (atrial fibrillation) models:
    a. Model fitted for M3 *given only SES and confounders*
    b. Model fitted for M3 *given SES, confounders, and M1.*
    c. Model fitted for M3 *given SES, confounders, and **M2**.*
    d. Model fitted for M3 *given SES, confounders, M1, and **M2**.*

4. **M4** ("stroke characteristics") models:
    a. Models fitted for each of the mediators in **M4** *given the SES and confounders, and any mediator in **M4** that precede in the sequence.*
    b. Models fitted for each of the mediators in **M4** *given the SES and confounders, any mediator in **M4** that precede in the sequence, M1, and **M2**.*

---

[1] https://github.com/anitalindmark/Interventions_SES_PROMs

c. Models fitted for each of the mediators in **M4** *given the SES and confounders, any mediator in **M4** that precede in the sequence, M1, and M3.*
d. Models fitted for each of the mediators in **M4** *given the SES and confounders, any mediator in **M4** that precede in the sequence, **M2**, and M3.*
e. Models fitted for each of the mediators in **M4** *given the SES and confounders, any mediator in **M4** that precede in the sequence, M1, **M2**, and M3.*

5. Outcome (poor PROMs) models:
   a. Models fitted for each outcome*, given SES, confounders, M1, **M2**, M3, and **M4**.*

Step B: Randomly draw mediator values and predict risks of poor PROMs:

In this step we randomly draw mediator values from binomial distributions with predicted probabilities from the models fitted in Step A (1.-4.). These randomly drawn values are then used to predict risks of poor outcome for each PROM for specific intervention scenarios from models 5.a. Note that different subsets of data are used for each contrast. For the low vs. mid SES contrast only patients with low and mid SES are used, and for the low vs. high SES contrast only patients with low and high SES are used.

Randomly draw mediator values
We randomly draw values from the *joint distribution of all mediators* under exposure (low SES) and no exposure (mid or high SES depending on the contrast of interest), respectively, as follows:

For each patient using the patient's observed confounder values and for each exposure setting draw

i. M1 using predicted probabilities from 1.a.,
ii. **M2** using predicted probabilities from 2.b. using the M1 value from i.
iii. M3 using predicted probabilities from 3.d. using the M1 value from i. and the **M2** values from ii.
iv. **M4** using predicted probabilities from 4.e., using the M1 value from i., the **M2** values from ii. and the M3 value from iii.

The random draws are repeated 500 times for each patient.

To simulate *single mediator interventions* mediator value(s) from the mediator/mediator group that is the target for intervention is generated from its distribution given only exposure and confounders, under no exposure (mid or high SES depending on the contrast of interest) while the other mediators are drawn from their joint distribution under exposure (low SES). Below this is exemplified for **M2**:

For each patient using the patient's observed confounder values:

I. Draw **M2 values** using predicted probabilities from 2.a. under no exposure.
II. Draw the other mediators from their joint distribution under exposure by first drawing M1 using predicted probabilities from 1.a., then drawing M3 using predicted probabilities from 3.b. and the drawn M1 value, and finally drawing **M4** using predicted probabilities from 4.c. and the drawn M1 and M3 values.

The random draws are repeated 500 times for each patient.

Predict risks of poor PROMs
For each of the 500 simulations, let:

$risk_{exposed}$ = Predicted risk of poor PROM under exposure (i.e. exposure set to low SES and mediators drawn from their joint distribution under low SES, Step i.-iv.), averaged over all patients.

$risk_{unexposed}$ = Predicted risk of poor PROM under no exposure (i.e. exposure set to mid (high) SES and mediators drawn from their joint distribution under mid (high) SES, Step i.-iv.), averaged over all patients.

$risk_{joint\_int}$ = Predicted risk of poor PROM under exposure (low SES) and mediators drawn from their joint distribution under no exposure (mid (high) SES, Step i.-iv.), averaged over all patients.

$risk_{\mathbf{M2}\_int}$ = Predicted risk of poor PROM under exposure (low SES) with **M2** drawn from its distribution under no exposure (mid (high) SES, Step I.), and the other mediators drawn from their joint distribution under exposure (low SES, Step II.) averaged over all patients.

Average predicted risks of poor PROMs under interventions on only M1, M3, and **M4**, respectively ($risk_{M1\_int}$, $risk_{M3\_int}$, and $risk_{\mathbf{M4}\_int}$) are defined similarly to $risk_{\mathbf{M2}\_int}$.

Step C: Effect estimation:

In this step the effects of interest are estimated based on contrasts of the predicted risks from Step B.

The effects are estimated by the following contrasts averaged over all simulations:

**Effect**	**Estimator, average*:**
Total risk difference	$risk_{exposed}$ - $risk_{unexposed}$
Joint intervention on all mediators:	
Reduction in total risk difference	$risk_{exposed}$ - $risk_{joint\_int}$
Remaining disparity	$risk_{joint\_int}$- $risk_{unexposed}$
Intervention on M1 alone	
Reduction in total risk difference	$risk_{exposed}$ - $risk_{M1\_int}$
Remaining disparity	$risk_{M1\_int}$ - $risk_{unexposed}$
*Intervention on **M2** alone*	
Reduction in total risk difference	$risk_{exposed}$ - $risk_{\mathbf{M2}\_int}$
Remaining disparity	$risk_{\mathbf{M2}\_int}$ - $risk_{unexposed}$
Intervention on M3 alone	
Reduction in total risk difference	$risk_{exposed}$ - $risk_{M3\_int}$
Remaining disparity	$risk_{M3\_int}$ - $risk_{unexposed}$
*Intervention on **M4** alone*	
Reduction in total risk difference	$risk_{exposed}$ - $risk_{\mathbf{M4}\_int}$
Remaining disparity	$risk_{\mathbf{M4}\_int}$ - $risk_{unexposed}$

* Over all 500 simulations

Standard errors of the effects are obtained through non-parametric bootstrap with 1000 bootstrap replications.

Table S1: Patient characteristics, number (%), separated by responders and non-responders to the 3 month follow up.

	Responders (n=7020)	Non-responders (n=1893)
**Socioeconomic status**		
Low	546 (7.8%)	262 (13.8%)
Mid	5350 (76.2%)	1356 (71.6%)
High	1014 (14.4%)	182 (9.6%)
Missing	110 (1.6%)	93 (4.9%)
**Sex: Male**	4528 (64.5%)	1284 (67.8%)
**Age,** Median [Q1,Q3]	57.0 [51.0,61.0]	55.0 [48.0,60.0]
**Smoking status**		
Smoker	1807 (25.7%)	599 (31.6%)
Non-smoker	4716 (67.2%)	1126 (59.5%)
Unknown	497 (7.1%)	168 (8.9%)
**Diabetes**		
Yes	1106 (15.8%)	333 (17.6%)
No	5910 (84.2%)	1559 (82.4%)
Missing	4 (0.1%)	1 (0.1%)
**Antihypertensives**		
Yes	2551 (36.3%)	681 (36.0%)
No	4451 (63.4%)	1206 (63.7%)
Missing	18 (0.3%)	6 (0.3%)
**Statins**		
Yes	1075 (15.3%)	250 (13.2%)
No	5927 (84.4%)	1631 (86.2%)
Missing	18 (0.3%)	12 (0.6%)
**Atrial fibrillation**		
Yes	629 (9.0%)	165 (8.7%)
No	6356 (90.5%)	1719 (90.8%)
Missing	35 (0.5%)	9 (0.5%)
**Stroke type**		
Hemorrhagic (I61)	942 (13.4%)	366 (19.3%)
Ischemic (I63)	6078 (86.6%)	1527 (80.7%)
**Lowered consciousness**		
Yes	522 (7.4%)	225 (11.9%)
No	6418 (91.4%)	1629 (86.1%)
Missing	80 (1.1%)	39 (2.1%)

Table S2: Exposure-outcome and mediator-outcome associations, estimated OR with 95% CI from logistic regression models with the different PROMs at 3 months as outcomes.

	ADL-dependency (n=6687)		Low mood (n=6510)		Fatigue (n=6600)		Pain (n=6537)		General health (n=6385)	
	Model 1[a]	*Model 2[b]*	*Model 1[a]*	*Model 2[b]*	*Model 1[a]*	*Model 2[b]*	*Model 1[a]*	*Model 2[b]*	*Model 1[a]*	*Model 2[b]*
Associations	*OR (95% CI)*	*OR (95% CI)*	*OR (95% CI)*	*OR (95% CI)*	*OR (95% CI)*	*OR (95% CI)*	*OR (95% CI)*	*OR (95% CI)*	*OR (95% CI)*	*OR (95% CI)*
Exposure—outcome model:										
**Low vs. mid SES—outcome**	1.94 (1.48-2.51)	1.64 (1.22-2.19)	2.19 (1.75-2.72)	1.87 (1.49-2.34)	1.30 (1.08-1.56)	1.20 (1.00-1.45)	1.47 (1.20-1.80)	1.32 (1.07-1.61)	1.97 (1.60-2.43)	1.70 (1.37-2.10)
**Low vs. high SES—outcome**	2.67 (1.87-3.83)	2.12 (1.43-3.16)	4.12 (3.02-5.66)	3.06 (2.22-4.25)	1.87 (1.50-2.33)	1.63 (1.30-2.05)	2.69 (2.08-3.49)	2.20 (1.69-2.87)	3.18 (2.41-4.20)	2.46 (1.85-3.27)
Mediator–outcome models:										
**Smoker—outcome**	1.00 (0.82–1.22)	-	1.82 (1.56–2.13)	-	1.23 (1.09 – 1.38)	-	1.34 (1.18-1.53)	-	1.43 (1.24–1.65)	-
**Metabolic health-outcome:**										
**Diabetes—outcome**	1.44 (1.15–1.78)	-	1.13 (0.92–1.37)	-	1.16 (1.01 – 1.33)	-	1.43 (1.23-1.66)	-	1.49 (1.26–1.75)	-
**Antihypertensives—outcome**	1.23 (1.02–1.47)	-	1.21 (1.03–1.41)	-	1.34 (1.20–1.49)	-	1.38 (1.23-1.56)	-	1.41 (1.23–1.61)	-
**Statins—outcome**	1.06 (0.83–1.34)	-	1.12 (0.92–1.37)	-	1.55 (1.35–1.78)	-	1.51 (1.29-1.76)	-	1.41 (1.19– 1.67)	-
**Atrial fibrillation—outcome**	1.28 (0.96–1.69)	-	1.19 (0.93–1.52)	-	1.05 (0.88–1.25)	-	1.05 (0.86-1.28)	-	1.31 (1.05–1.62)	-
**Stroke characteristics- outcome:**										
**Hemorrhagic vs. ischemic stroke-outcome**	3.23 (2.64–3.94)	-	0.95 (0.76–1.18)	-	1.01 (0.87–1.17)	-	1.06 (0.89-1.25)	-	1.13 (0.93–1.36)	-
**Lowered consciousness-outcome**	12.38 (10.01–15.30)	-	1.81 (1.42–2.30)	-	1.21 (0.99–1.46)	-	1.31 (1.05-1.62)	-	2.05 (1.64–2.55)	-

ADL=Activities of daily living; SES=socioeconomic status

[a] Adjusted for confounders sex, age, age-squared, and for mediator-outcome models the exposure socioeconomic status.

[b] Adjusted for confounders and mediators.

Table S3: Mediator-outcome associations, estimated OR with 95% CI from logistic regression models adjusted for the confounders sex, age, and age-squared (based on the total study population, n=6910).

Associations	***OR***	***95% CI***
Exposure–mediator models:		
**SES-smoking status:**		
Low vs. mid SES—Smoker[a]	2.27	1.89 – 2.71
Low vs. high SES—Smoker[a]	8.75	6.66 – 11.57
**SES-metabolic health:**		
Low vs. mid SES—diabetes	1.95	1.58 – 2.39
Low vs. high SES—diabetes	2.73	2.07 – 3.59
Low vs. mid SES—antihypertensives	1.50	1.24 – 1.80
Low vs. high SES—antihypertensives	2.00	1.60 – 2.50
Low vs. mid SES—statins	1.29	1.02 – 1.62
Low vs. high SES—statins	1.61	1.21 – 2.14
**SES-atrial fibrillation:**		
Low vs. mid SES—atrial fibrillation	0.96	0.69 – 1.30
Low vs. high SES—atrial fibrillation	1.03	0.71 – 1.49
**SES-stroke characteristics:**		
Low vs. mid SES—hemorrhagic stroke[b]	0.93	0.71 – 1.21
Low vs. high SES—hemorrhagic stroke[b]	0.88	0.64 – 1.19
Low vs. mid SES—lowered consciousness	1.56	1.16 – 2.07
Low vs. high SES—lowered consciousness	1.78	1.23 – 2.58

SES=Socioeconomic status

[a]Smoker vs. non-smoker/unknown.

[b]Hemorrhagic vs. ischemic stroke.

Table S4: Results from mediation analysis for the outcome dependency in activities of daily living (ADL) at 3 months. Estimated absolute risk differences (%) based on 500 Monte Carlo simulations with standard errors based on 1000 bootstrap replicates.

	Low vs. mid SES				***Low vs. high SES***			
	**Absolute risk difference (95% CI)**	**P-value**	**Proportion reduced[a] (%)**	**Remaining disparity (95% CI)**	**Absolute risk difference (95% CI)**	**P-value**	**Proportion reduced[a] (%)**	**Remaining disparity (95% CI)**
**Total adjusted risk difference**	6.5 (3.3, 9.6)	<0.001		-	8.4 (5.0, 11.8)	<0.001		-
**Risk reduction from intervening on:**								
**All mediators**	1.9 (-0.1, 4.0)	0.067	29.7	4.5 (1.3, 7.7)	2.3 (-0.8, 5.3)	0.146	26.7	6.2 (2.1, 10.2)
**Smoking status**	-0.2 (-1.2, 0.7)	0.630	-3.7	6.7 (3.3, 10.1)	-0.4 (-2.4, 1.6)	0.726	-4.2	8.8 (4.7, 12.9)
**Metabolic health**	0.7 (-0.3, 1.6)	0.156	10.6	5.8 (2.6, 8.9)	0.9 (-0.3, 2.2)	0.150	11.2	7.5 (4.0, 11.0)
**Atrial fibrillation**	-0.1 (-0.5, 0.4)	0.805	-0.8	6.5 (3.3, 9.7)	-0.0 (-0.5, 0.4)	0.927	-0.2	8.5 (5.1, 11.9)
**Stroke characteristics**	1.6 (0.2, 3.0)	0.028	24.5	4.9 (2.1, 7.6)	1.8 (0.1, 3.4)	0.032	20.9	6.7 (3.7, 9.7)

[a] (Risk reduction)/(Total adjusted risk difference)*100.

Table S5: Results from mediation analysis for the outcome low mood often/constantly at 3 months. Estimated absolute risk differences (%) based on 500 Monte Carlo simulations with standard errors based on 1000 bootstrap replicates.

	Low vs. mid SES				***Low vs. high SES***			
	**Absolute risk difference (95% CI)**	**P-value**	**Proportion reduced[a] (%)**	**Remaining disparity (95% CI)**	**Absolute risk difference (95% CI)**	**P-value**	**Proportion reduced[a] (%)**	**Remaining disparity (95% CI)**
**Total adjusted risk difference**	11.7 (7.7, 15.6)	<0.001		-	17.4 (13.2, 21.6)	<0.001		-
**Risk reduction from intervening on:**								
**All mediators**	1.7 (-0.4, 3.7)	0.120	14.2	10.0 (5.7, 14.3)	3.4 (0.0, 6.8)	0.050	19.5	14.0 (8.8, 19.1)
**Smoking status**	1.4 (-0.0, 2.8)	0.051	12.0	10.3 (6.2, 14.3)	2.7 (0.0, 5.3)	0.047	15.4	14.7 (9.9, 19.5)
**Metabolic health**	-0.0 (-1.2, 1.1)	0.970	-0.2	11.7 (7.6, 15.8)	0.3 (-1.3, 1.8)	0.754	1.5	17.1 (12.6, 21.6)
**Atrial fibrillation**	-0.0 (-0.6, 0.5)	0.860	-0.4	11.7 (7.8, 15.7)	-0.0 (-0.6, 0.5)	0.873	-0.3	17.4 (13.2, 21.6)
**Stroke characteristics**	0.3 (-0.6, 1.2)	0.510	2.5	11.4 (7.5, 15.2)	0.3 (-0.7, 1.3)	0.589	1.6	17.1 (12.9, 21.2)

[a] (Risk reduction)/(Total adjusted risk difference)*100.

Table S6: Results from mediation analysis for the outcome fatigue often/constantly at 3 months. Estimated absolute risk differences (%) based on 500 Monte Carlo simulations with standard errors based on 1000 bootstrap replicates.

	Low vs. mid SES				***Low vs. high SES***			
	**Absolute risk difference (95% CI)**	**P-value**	**Proportion reduced[a] (%)**	**Remaining disparity (95% CI)**	**Absolute risk difference (95% CI)**	**P-value**	**Proportion reduced[a] (%)**	**Remaining disparity (95% CI)**
**Total adjusted risk difference**	6.2 (1.6, 10.8)	0.008		-	14.9 (9.5, 20.3)	<0.001		-
**Risk reduction from intervening on:**								
**All mediators**	2.8 (0.3, 5.2)	0.026	44.6	3.5 (-1.5, 8.4)	5.4 (1.3, 9.6)	0.010	36.6	9.4 (2.9, 15.9)
**Smoking status**	1.7 (0.0, 3.4)	0.057	26.5	4.6 (-0.2, 9.4)	3.3 (0.1, 6.4)	0.043	22.0	11.6 (5.4, 17.8)
**Metabolic health**	1.1 (-0.3, 2.6)	0.129	18.2	5.1 (0.4, 9.8)	2.0 (-0.1, 4.1)	0.064	13.3	12.9 (7.3, 18.5)
**Atrial fibrillation**	-0.1 (-0.6, 0.4)	0.638	-1.9	6.4 (1.8, 11.0)	-0.1 (-0.7, 0.4)	0.665	-0.8	15.0 (9.6, 20.4)
**Stroke characteristics**	0.0 (-0.7, 0.8)	0.929	0.6	6.2 (1.6, 10.8)	0.1 (-0.9, 1.0)	0.892	0.4	14.8 (9.4, 20.2)

[a] (Risk reduction)/(Total adjusted risk difference)*100.

Table S7: Results from mediation analysis for the outcome pain often/constantly at 3 months. Estimated absolute risk differences (%) based on 500 Monte Carlo simulations with standard errors based on 1000 bootstrap replicates.

	Low vs. mid SES				***Low vs. high SES***			
	**Absolute risk difference (95% CI)**	**P-value**	**Proportion reduced[a] (%)**	**Remaining disparity (95% CI)**	**Absolute risk difference (95% CI)**	**P-value**	**Proportion reduced[a] (%)**	**Remaining disparity (95% CI)**
**Total adjusted risk difference**	7.6 (3.5, 11.8)	<0.001		-	17.3 (12.8, 21.8)	<0.001		-
**Risk reduction from intervening on:**								
**All mediators**	2.4 (0.1, 4.7)	0.041	31.3	5.3 (0.7, 9.8)	4.6 (0.9, 8.4)	0.016	26.8	12.7 (7.0, 18.4)
**Smoking status**	1.2 (-0.5, 2.8)	0.159	15.3	6.5 (2.0, 10.9)	2.3 (-0.8, 5.3)	0.147	13.1	15.0 (9.6, 20.5)
**Metabolic health**	1.4 (0.0, 2.8)	0.050	18.4	6.2 (2.0, 10.5)	2.3 (0.3, 4.2)	0.022	13.2	15.0 (10.3, 19.8)
**Atrial fibrillation**	0.2 (-0.4, 0.8)	0.567	2.4	7.5 (3.3, 11.6)	0.3 (-0.4, 1.0)	0.423	1.7	17.0 (12.5, 21.5)
**Stroke characteristics**	0.1 (-0.5, 0.6)	0.775	1.1	7.6 (3.4, 11.7)	0.1 (-0.6, 0.8)	0.858	0.4	17.2 (12.7, 21.8)

[a] (Risk reduction)/(Total adjusted risk difference)*100.

Table S8: Results from mediation analysis for the outcome self-rated general health quite poor/very poor at 3 months. Estimated absolute risk differences (%) based on 500 Monte Carlo simulations with standard errors based on 1000 bootstrap replicates.

	Low vs. mid SES				***Low vs. high SES***			
	**Absolute risk difference (95% CI)**	**P-value**	**Proportion reduced[a] (%)**	**Remaining disparity (95% CI)**	**Absolute risk difference (95% CI)**	**P-value**	**Proportion reduced[a] (%)**	**Remaining disparity (95% CI)**
**Total adjusted risk difference**	12.2 (7.9, 16.6)	<0.001		-	18.2 (13.5, 22.9)	<0.001		-
**Risk reduction from intervening on:**								
**All mediators**	3.5 (1.1, 5.9)	0.004	28.6	8.7 (4.2, 13.3)	6.3 (2.6, 10.1)	0.001	34.8	11.9 (6.4, 17.3)
**Smoking status**	2.2 (0.5, 3.8)	0.009	17.7	10.1 (5.6, 14.5)	4.2 (1.3, 7.2)	0.005	23.1	14.0 (8.7, 19.3)
**Metabolic health**	1.2 (-0.2, 2.6)	0.101	9.7	11.0 (6.7, 15.4)	1.7 (-0.3, 3.6)	0.098	9.2	16.5 (11.7, 21.4)
**Atrial fibrillation**	0.3 (-0.5, 1.0)	0.522	2.1	12.0 (7.6, 16.3)	0.4 (-0.5, 1.2)	0.359	2.2	17.8 (13.1, 22.5)
**Stroke characteristics**	0.5 (-0.3, 1.4)	0.236	4.2	11.7 (7.4, 16.0)	0.5 (-0.5, 1.5)	0.299	2.8	17.7 (13.0, 22.4)

[a] (Risk reduction)/(Total adjusted risk difference)*100.

Table S9: Descriptive statistics comparing level of consciousness and NIHSS.

	Level of consciousness			
	**Fully conscious (n=6320)**	**Lowered consiousness (n=513)**	**Missing (n=77)**	**Overall (n=6910)**
**NIHSS**				
Mean (SD)	3.42 (4.53)	13.6 (7.40)	9.46 (6.97)	4.04 (5.33)
Median [Q1, Q3]	2.00 [0, 4.0]	15.0 [7.0, 19.0]	9.00 [2.75, 14.3]	2.00 [0, 5.0]
Missing	2387 (37.8%)	273 (53.2%)	53 (68.8%)	2713 (39.3%)

NIHSS=National Institutes of Health Stroke Scale; SD=Standard deviation.

Table S10: Logistic regression models for the outcomes poor PROMs among patients with both level of consciousness and NIHSS observed. Adjusted for sex, age, and age-squared, and without (Model A) and with (Model B) adjustment for NIHSS score.

	**ADL-dependency (n=4094)**		**Low mood (n=3985)**		**Fatigue (n=4050)**		**Pain (n=4015)**		**General health (n=3915)**	
	Model A	*Model B*	*Model A*	*Model B*	*Model A*	*Model B*	*Model A*	*Model B*	*Model A*	*Model B*
	OR (95% CI)	*OR (95% CI)*	*OR (95% CI)*	*OR (95% CI)*	*OR (95% CI)*	*OR (95% CI)*	*OR (95% CI)*	*OR (95% CI)*	*OR (95% CI)*	*OR (95% CI)*
**Low vs. mid SES**	1.32 (0.86 – 1.97)	1.60 (1.03 – 2.42)	1.81 (1.35 – 2.42)	1.83 (1.36 – 2.44)	1.05 (0.82 – 1.34)	1.05 (0.82 – 1.34)	1.30 (0.99 – 1.69)	1.33 (1.01 – 1.73)	1.52 (1.14 – 2.01)	1.55 (1.16 – 2.06)
**Low vs. high SES**	1.45 (0.85 – 2.45)	1.51 (0.87 – 2.60)	2.61 (1.75 – 3.91)	2.59 (1.74 – 3.88)	1.44 (1.07 – 1.92)	1.43 (1.07 – 1.92)	1.99 (1.41 – 2.79)	1.98 (1.41 – 2.79)	2.11 (1.46 – 3.04)	2.08 (1.44 – 3.02)
**Smoker**	1.11 (0.83 – 1.46)	1.13 (0.83 – 1.51)	1.87 (1.53 – 2.28)	1.86 (1.53 – 2.27)	1.18 (1.02 – 1.37)	1.18 (1.02 – 1.37)	1.38 (1.16 – 1.63)	1.36 (1.15 – 1.61)	1.45 (1.20 – 1.74)	1.43 (1.19 – 1.72)
**Diabetes**	1.80 (1.28 – 2.51)	2.15 (1.51 – 3.05)	1.17 (0.89 – 1.54)	1.19 (0.90 – 1.56)	0.96 (0.78 – 1.17)	0.96 (0.79 – 1.17)	1.28 (1.02 – 1.59)	1.29 (1.03 – 1.61)	1.40 (1.10 – 1.77)	1.43 (1.12 – 1.81)
**Antihypertensives**	1.27 (0.96 – 1.67)	1.25 (0.93 – 1.67)	1.28 (1.04 – 1.59)	1.28 (1.03 – 1.58)	1.23 (1.06 – 1.43)	1.23 (1.06 – 1.43)	1.14 (0.96 – 1.35)	1.13 (0.95 – 1.34)	1.19 (0.98 – 1.44)	1.18 (0.97 – 1.43)
**Statins**	1.01 (0.70 – 1.44)	1.03 (0.70 – 1.50)	0.94 (0.70 – 1.25)	0.94 (0.70 – 1.26)	1.50 (1.23 – 1.83)	1.51 (1.24 – 1.84)	1.43 (1.14 – 1.78)	1.44 (1.15 – 1.80)	1.24 (0.97 – 1.58)	1.26 (0.98 – 1.61)
**Atrial fibrillation**	1.20 (0.79 – 1.76)	0.96 (0.63 – 1.43)	1.24 (0.90 – 1.68)	1.19 (0.86 – 1.61)	1.06 (0.85 – 1.32)	1.05 (0.84 – 1.31)	1.07 (0.82 – 1.37)	1.00 (0.77 – 1.29)	1.47 (1.12 – 1.92)	1.38 (1.05 – 1.80)
**Hemorrhagic vs. ischemic stroke**	1.93 (1.37 – 2.68)	1.36 (0.95 – 1.93)	0.83 (0.59 – 1.15)	0.79 (0.55 – 1.10)	0.96 (0.77 – 1.20)	0.95 (0.76 – 1.18)	1.01 (0.78 – 1.30)	0.92 (0.71 – 1.19)	1.01 (0.75 – 1.34)	0.91 (0.68 – 1.22)
**Lowered consciousness**	10.35 (7.58 – 14.10)	2.75 (1.89 – 3.97)	1.92 (1.35 – 2.69)	1.49 (1.00 – 2.19)	1.16 (0.88 – 1.53)	1.08 (0.79 – 1.47)	1.72 (1.27 – 2.32)	1.17 (0.83 – 1.64)	2.10 (1.52 – 2.88)	1.33 (0.92 – 1.91)
**NIHSS**	-	1.16 (1.14 – 1.18)	-	1.03 (1.01 – 1.05)	-	1.01 (0.99 – 1.02)	-	1.04 (1.02 – 1.06)	-	1.05 (1.03 – 1.06)

ADL=Activities of daily living; SES=socioeconomic status; NIHSS=National Institutes of Health Stroke Scale.
